# Editorial: Type 2 inflammatory skin diseases: novel therapies and clinical insights

**DOI:** 10.3389/fmed.2026.1879881

**Published:** 2026-06-18

**Authors:** Linfeng Li

**Affiliations:** Department of Dermatology, Beijing Friendship Hospital, Capital Medical University, Beijing, China

**Keywords:** atopic dermatitis, IgE, skin, type 2 inflammatory diseases, urticaria

Type 2 inflammatory skin diseases are a hot topic in dermatology research. The most common of these is atopic dermatitis. With the advent of innovative targeted therapies, including monoclonal antibodies against IL-4Rα, IL-5, IL-13, IL-31, JAK inhibitors, and PDE-4 inhibitors, more and more type 2 inflammatory skin diseases have been reported, such as bullous pemphigoid, prurigo nodularis, chronic spontaneous urticaria, alopecia areata, hypereosinophilic syndrome, hyperimmunoglobulin E syndrome, and pruritus. Type 2 inflammation is an abnormal type 2 immune response that is primarily anti-parasitic. Eosinophils and IgE antibodies have very strong anti-parasitic effects. The occurrence of type 2 inflammation can be divided into several stages. Both genetic and external environment factors can damage skin barrier function, leading to the penetration of environmental allergens, irritants, and microorganisms into the skin and stimulating epithelial cells to produce alarmins (TSLP, IL-23, IL-33, etc.). Alarmins act on group 2 innate lymphoid cells, CD4+Th2 cells, and CD8+TC2 cells to produce IL-4, 5, 9, 13, and 31, among others, which mediate type 2 inflammation. Meanwhile, the immune system shifts toward the production of IgE, leading to an increase in total IgE and the generation of allergen-specific IgE, further exacerbating type 2 inflammation.

This Research Topic aims to consolidate and expand our current understanding of the pathogenesis and management of Type 2 inflammatory skin diseases. First of all, many clinicians are interested in comparing biologics and JAK inhibitors in the treatment of atopic dermatitis. Prados-Carmona et al. conducted a real-world study in southern Spain comparing biologics and JAK inhibitors in moderate-to-severe atopic dermatitis. Their study used two clinically and demographically comparable patient cohorts. Treatment outcomes were assessed at short-term (16 weeks) and medium-to-long-term (24–52 weeks) intervals, revealing that JAK inhibitors produced faster early responses, while biologics achieved greater overall improvements between weeks 16 and 52, in addition to longer treatment persistence over time. Similar super-responder rates were observed across all groups. Both treatment classes demonstrated favorable safety profiles, with different patterns of adverse events, supporting a tailored approach to therapy selection in routine clinical practice. Recently, concern has been raised about whether dupilumab treatment will increase the risk of lymphoma. Kridin et al. studied the association between AD and lymphoma risk and extended it to non-dermatological type 2 inflammatory diseases (T2IDs) along with the impact of dupilumab on lymphoma risk in AD and non-dermatological T2IDs by conducting a retrospective cohort study. They found that among 801,508 cases and controls, AD was associated with an increased risk of lymphoma, including cutaneous T-cell lymphoma (CTCL) and non-Hodgkin lymphoma (NHL). Among 14.4 million cases and controls, non-dermatological T2IDs were also associated with an increased lymphoma risk. In the comparison of AD patients treated with dupilumab with other systemic treatments (*n* = 7,840 per group), dupilumab exposure was not linked to an increased lymphoma risk, although it tended to reduce it. This decreased risk association was most evident in non-dermatological T2IDs (*n* = 16,908 per group). The authors concluded that T2IDs, including AD, are associated with a significantly increased risk of lymphoma. Treatment with dupilumab partially ameliorates this risk association, especially for NHL.

Although biologics and small molecules have achieved certain therapeutic effects in the treatment of T2IDs, they still cannot solve the issue of recurrence. Therefore, further research is needed. Liu et al. discussed molecular insights into the role of vitamin D in atopic dermatitis with regard to pathogenesis, diagnosis, and emerging therapies. Tian et al. suggested that platelet-rich plasma is an adjunct therapy for eczema, targeting inflammation, skin barrier repair, and chronic recurrence. Tong et al. conducted a systematic review and meta-analysis to evaluate the efficacy and safety of acupuncture in the treatment of chronic spontaneous urticaria (CSU).

Although allergen-specific IgE is not a prerequisite for type 2 inflammation, its presence is a biological marker for this condition. Therefore, the detection of allergen-specific IgE is very important. Błażowska et al. discussed the significance of component-resolved diagnostics in atopic dermatitis. Component-resolved diagnostics (CRD) offer sIgE testing of individual allergen molecules and provide additional insights, especially in polysensitized patients, in case of sensitization to allergens with low abundance, low stability, or associated with risks of anaphylaxis. It enables the detection of genuine and cross-sensitization or the composition of an allergen immunotherapy vaccine. The utility of CRD has never been thoroughly analyzed before. This review provides basic information about CRD and comprehensively summarizes its potential applications in the personalized management of patients with atopic dermatitis. Molecular profiling of allergen components is helping to explain the mechanisms by which different molecular endotypes and clinical phenotypes of atopic dermatitis are developed, and is providing biomarkers of disease severity, autoimmune IgE responses, and therapeutic response, improving our understanding of atopic dermatitis endotypes and treatment outcomes.

In summary, the articles published in this Research Topic cover various aspects of Type 2 inflammatory skin diseases. They range from case reports to reviews and basic and clinical original studies. As more and more skin diseases are found to be related to Type 2 inflammation, the study of Type 2 inflammatory skin diseases is crucial and demands more research.

